# Author Correction: Evidence for trivial Berry phase and absence of chiral anomaly in semimetal NbP

**DOI:** 10.1038/s41598-018-32404-3

**Published:** 2018-10-16

**Authors:** Sudesh, Pawan Kumar, Prakriti Neha, Tanmoy Das, Satyabrata Patnaik

**Affiliations:** 1 School of Physical Sciences, Jawaharlal Nehru University, New Delhi-110067, India; 2 Department of Physics, Indian Institute of Science, Bengalore-560012, India

Correction to: *Scientific Reports* 10.1038/srep46062, published online 10 April 2017

The Acknowledgements section in this Article is incomplete.

“Sudesh, P. Kumar and P. Neha acknowledge UGC-D. S. Kothari fellowship, JNU (New Delhi) and UGC-BSR, respectively for financial support. Authors are thankful to AIRF (JNU) for access to the PPMS, EDAX and TEM facilities. We thank Prof A. K. Rastogi and Dr. Chandra Shekhar for useful discussions. Low–temperature high magnetic field at JNU is supported under the FIST program of Department of Science and Technology, Government of India. T. Das’s work is supported by SERB young scientist Startup research grant. We thank Dr. Dinabandhu Das for advice on single crystal diffraction data analysis.”

should read:

“Sudesh, P. Kumar and P. Neha acknowledge UGC-D. S. Kothari fellowship, JNU (New Delhi) and UGC-BSR, respectively for financial support. Authors are thankful to AIRF (JNU) for access to the PPMS, EDAX and TEM facilities. We thank Prof A. K. Rastogi and Dr. Chandra Shekhar for useful discussions. Low–temperature high magnetic field at JNU is supported under the FIST program of Department of Science and Technology, Government of India. We also acknowledge financial support received from DST-PURSE grant (Government of India) to fund the open access charges for this manuscript. T. Das’s work is supported by SERB young scientist Startup research grant. We thank Dr. Dinabandhu Das for advice on single crystal diffraction data analysis.”

